# Unusual presentation of prune belly syndrome: a case report

**DOI:** 10.1186/s13256-017-1487-9

**Published:** 2017-12-04

**Authors:** Abayneh Girma Demisse, Ashenafi Berhanu, Temesgen Tadesse

**Affiliations:** 1 Department of Pediatrics and Child Health, School of Medicine, College of Medicine and Health Science, University of Gondar, Gondar, Ethiopia; 2 Department of Surgery, School of Medicine, College of Medicine and Health Science, University of Gondar, Gondar, Ethiopia; 3 Department of Radiology, School of Medicine, College of Medicine and Health Science, University of Gondar, Gondar, Ethiopia

**Keywords:** Megacystis, Hip dysplasia, Undescended testes

## Abstract

**Background:**

Prune belly syndrome is a rare congenital malformation of unknown etiology, with the following triad of findings: abdominal muscle wall weakness, undescended testes, and urinary tract abnormalities. In most cases, detection of prune belly syndrome occurs during neonatal or infancy period. In this case report, we describe a 12-year-old boy from Ethiopia with the triad of findings of prune belly syndrome along with skeletal malformations. We are unaware of any previous report of prune belly syndrome in Ethiopia.

**Case presentation:**

A 12-year-old Amhara boy from the Northwest Gondar Amhara regional state presented to our referral hospital with a complaint of swelling over his left flank for the past 3 months. Maternal pregnancy course and medical history were noncontributory, and he had an attended birth at a health center. He has seven siblings, none of whom had similar symptoms. On examination he had a distended abdomen, asymmetric with bulging left flank, visible horizontal line, upward umbilical slit, and absent rectus abdominis muscles. His abdomen was soft with a tender cystic, bimanually palpable mass on the left flank measuring 13 × 11 cm. Both testes were undescended and he also has developmental dysplasia of the hips. An abdominal ultrasound revealed a large cystic mass in his left kidney area with echo debris and a hip X-ray showed bilateral developmental dysplasia of the hip.

Intraoperative findings were cystic left kidney, both testes were intraperitoneal, tortuous left renal vein, enlarged bladder reaching above umbilicus, and left megaureter.

Interventions: bilateral orchidectomy and left nephrectomy were done. He was given intravenously administered antibiotics for treatment of pyelonephritis and discharged home with an appointment for follow up and possible abdominoplasty.

**Conclusions:**

In the current report delayed presentation contributed to testicular atrophy and decision for orchidectomy. Furthermore, he will be at potential risk for sex hormone abnormality. Therefore, diagnosis of prune belly syndrome in resource-limited settings requires a high index of suspicion. We recommend further research to determine the optimal management and early diagnosis of prune belly syndrome in resource-limited settings.

## Background

Prune belly syndrome (PBS) is a rare multisystem disease characterized by a deficiency of abdominal wall musculature, bilateral intra-abdominal testes, and urinary tract abnormalities, including megacystis, hydroureteronephrosis, and renal dysplasia. PBS predominantly affects boys, with a contemporary incidence of 3.6 to 3.8 per 100,000 live births in the United States of America [1, 2]. There is wide variability in disease severity as PBS is not infrequently accompanied by cardiopulmonary, gastrointestinal, or musculoskeletal anomalies [3, 4]. There is no proven etiology for the development of PBS but a defect in mesodermal differentiation of the anterior abdominal wall and urinary tract between the 6th and 10th weeks of gestation has been suggested [5, 6].

## Case presentation

A 12-year-old Amhara boy from the Northwest Gondar Amhara regional state complained of left flank swelling for the last 3 months. Three days before admission he started to experience pain in his left flank, which was associated with high grade fever, chills, rigor, and vomiting. At the time he had no urinary or respiratory symptoms. Maternal pregnancy course and medical history were noncontributory, and he had an attended birth at a health center; his past medical history was unremarkable. He has seven siblings, none of whom had similar symptoms. His mother had no history of radiation exposure, use of traditional medicine, or chemotherapy during pregnancy.

### Physical examination

**Fig. 1:**
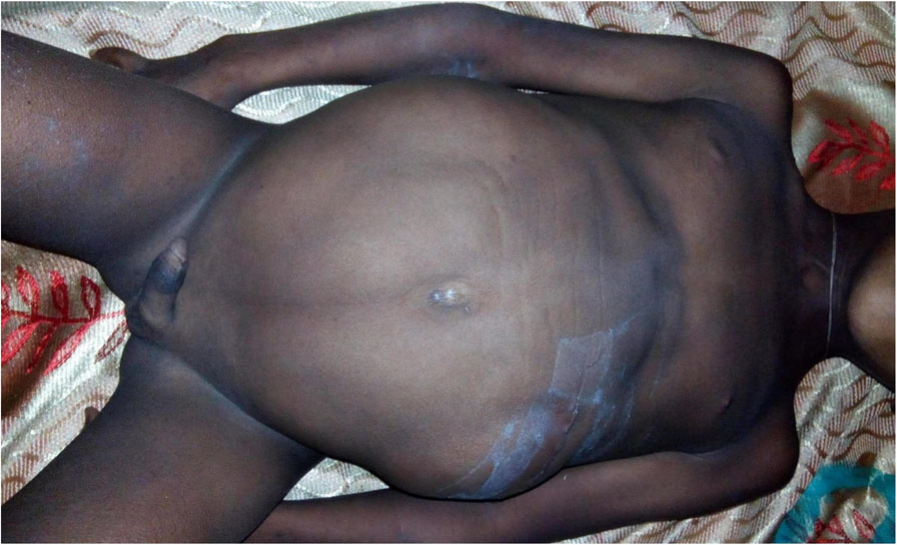
A 12-year-old boy with prune belly syndrome with empty scrotum and absent abdominal wall musculature

No dysmorphisms were noted in his general appearance. His vital signs were: pulse rate (PR) 142 beats/min, respiratory rate (RR) 33 breaths/min, temperature (T) 38.5 °C, and blood pressure (BP) 105/65 mmHg. Anthropometric measurements showed severe stunting. An abdominal examination revealed distended abdomen, asymmetric with bulging left flank, visible horizontal line, upward umbilical slit, and absent rectus abdominis muscles. His abdomen was soft with a tender cystic, bimanually palpable mass on the left flank measuring 13 × 11 cm. Both testes were undescended (Fig. 1). He also had scoliosis and developmental dysplasia of the hips with waddling gate.

### Investigations

**Fig. 2:**
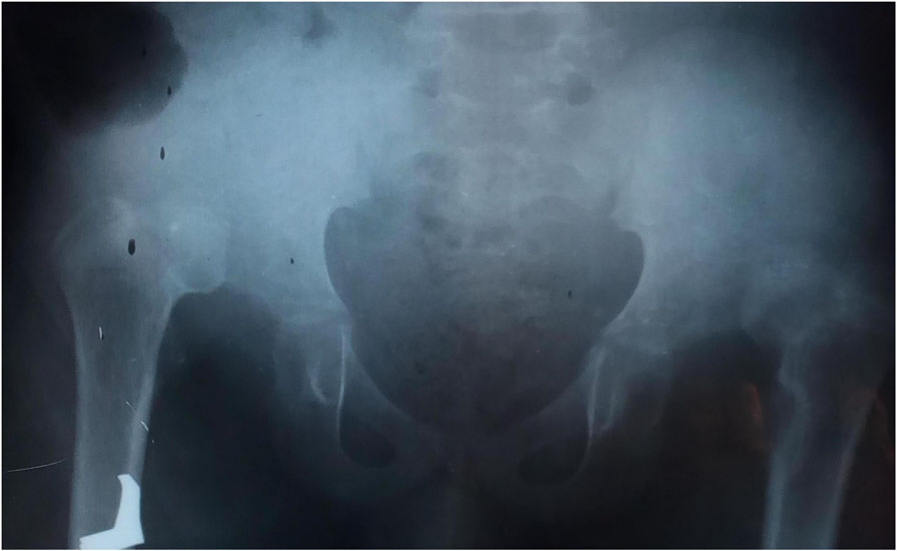
Pelvic X-ray of a 12-year-old boy with bilateral hip dysplasia

Urinary analysis demonstrated many red blood cells (RBCs) and positive leukocyte esterase. He had a normal renal function test with creatinine of 0.72 mg/dl and blood urea nitrogen (BUN) of 44 mg/dl, normal hemoglobin of 12.7 gm/dl, and normal electrolyte. Ultrasound showed a large cystic mass in his left kidney area with echo debris. His right kidney had normal echo texture and size. An X-ray of his hips showed bilateral developmental dysplasia of the hip (DDH; Fig. 2). Our facility does not have the capacity to perform a genetic study to support the diagnosis of PBS.

### Intraoperative findings

**Fig. 3:**
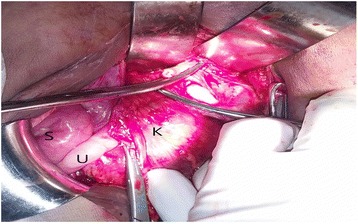
Intraoperative findings of a 12-year-old boy. *K* kidney, *S* small intestine, *U* ureter

His left kidney is cystic and enlarged; there is no parenchyma tissue, both testes are intraperitoneal just below the kidneys, tortuous left renal vein, enlarged bladder reaching above umbilicus, and left megaureter (Fig. 3). A bilateral orchidectomy and left nephrectomy were done.

### Course of our patient in our hospital

After surgical removal of cystic mass and testes, he was given intravenously administered antibiotics for treatment of pyelonephritis for 14 days. Subsequently he showed significant improvement and was discharged home with an appointment for follow up to consider hormonal replacement therapy and possible abdominoplasty. His parents were also counseled about the PBS and the care they can give him at home.

## Discussion

PBS is a rare congenital disorder composed of anomalies of various organ systems. This case report describes a 12-year-old boy with the triad findings of PBS reported in previous publications [7, 8] and congenital bilateral hip dysplasia with scoliosis. In the majority of case reports the diagnosis of PBS is made during infancy. Although this child was born at a health center his congenital malformation was not detected at birth. This may indicate a low level of awareness among health professionals about PBS; however, there are very few case reports of PBS diagnosed outside the neonatal age group [9]. The embryologic characteristics of congenital musculoskeletal problems correlate well with the embryologic theory of PBS [10]. Similar to other case reports, scoliosis and hip dysplasia were noted in the current case. Orthopedic abnormalities occur in 45 to 63% of patients with PBS [11]. The most common orthopedic anomalies are hip dislocation and talipes equinovarus [12], while scoliosis is the most common spinal abnormality noted [13, 14]. Currently, children with PBS reach adulthood; however, morbidity related to the musculoskeletal system can potentially affect their quality of life. The abdominal wall defect may not have life-threatening consequences but the inefficient contraction of the abdominal wall muscles is believed to affect bladder, bowel, and pulmonary function, increasing the risk of recurrent urinary and respiratory tract infections [15]. Improvement in bladder emptying is a potential benefit of offering abdominoplasty in full-blown PBS [16]. The incidence of renal failure over time was 20 to 30%, although some case series have rates of renal insufficiency as low as 7% [17]. In the current case, despite a non-functioning left kidney, the renal function test was within normal limits for our patient’s age, which could be explained by the compensatory action of his right kidney.

Treatment of the case involved both surgical and medical teams. Removal of our patient’s left kidney and orchidectomy were performed, and he was treated for a urinary tract infection. There have been documented cases of testicular tumors developing in these patients, but overall the risk does not appear to be greater than other patients with undescended testes [18–20]. Orchidopexy or removal of the testes is one of the treatment modalities available to children with PBS. Removal of the testes is done to counteract the high risk of cancer associated with intraperitoneal testes which was a potential outcome in this case.

He was discharged from our hospital with the expectation of long-term follow up, and an appointment for abdominoplasty was scheduled. After communicating with an endocrinologist, hormonal replacement therapy was considered to be started on follow up.

Our strength to the approach of this case was giving the most treatment and care that were available in our setting. In contrast, the unavailability of genetic study was our limitation.

## Conclusions

Diagnosis of PBS in resource-limited settings requires a high index of suspicion. Early diagnosis of PBS will help health professionals to carry out the appropriate steps of management and follow up. As the disease involves multiple organ systems, a multidisciplinary management approach is required to improve the quality of life for patients with PBS. We recommend further research to determine the optimal management and early diagnosis of PBS in resource-limited settings.

## Abbreviations

BP: Blood pressure
BUN: Blood urea nitrogen
DDH: Developmental dysplasia of the hip
PBS: Prune belly syndrome
PR: Pulse rate
RBC: Red blood cell
RR: Respiratory rate
T: Temperature
